# Metabolomic Profiling of *Nicotiana* Spp. Nectars Indicate That Pollinator Feeding Preference Is a Stronger Determinant Than Plant Phylogenetics in Shaping Nectar Diversity

**DOI:** 10.3390/metabo10050214

**Published:** 2020-05-22

**Authors:** Fredy A. Silva, Elizabeth C. Chatt, Siti-Nabilla Mahalim, Adel Guirgis, Xingche Guo, Daniel S. Nettleton, Basil J. Nikolau, Robert W. Thornburg

**Affiliations:** 1 Roy J Carver Department of Biochemistry, Biophysics and Molecular Biology, Iowa State University, Ames, IA 50011-1079, USA; fredydavi@gmail.com (F.A.S.); ecchatt@iastate.edu (E.C.C.); nuraziatull@ymail.com (S.-N.M.); adel1250eg@yahoo.com (A.G.); dimmas@iastate.edu (B.J.N.); 2 Department of Molecular Biology, Genetic Engineering and Biotechnology Research Institute, University of Sadat City, Sadat City 32958, Egypt; 3 Department of Statistics, Iowa State University, Ames, IA 50011-1079, USA; xguo@iastate.edu (X.G.); dnett@iastate.edu (D.S.N.)

**Keywords:** nectar, *Nicotiana*, phylogeny, plant-animal interactions, pollinators

## Abstract

Floral nectar is a rich secretion produced by the nectary gland and is offered as reward to attract pollinators leading to improved seed set. Nectars are composed of a complex mixture of sugars, amino acids, proteins, vitamins, lipids, organic and inorganic acids. This composition is influenced by several factors, including floral morphology, mechanism of nectar secretion, time of flowering, and visitation by pollinators. The objective of this study was to determine the contributions of flowering time, plant phylogeny, and pollinator selection on nectar composition in *Nicotiana*. The main classes of nectar metabolites (sugars and amino acids) were quantified using gas chromatography/mass spectrometric analytical platforms to identify differences among fifteen *Nicotiana* species representing day- and night-flowering plants from ten sections of the genus that are visited by five different primary pollinators. The nectar metabolomes of different *Nicotiana* species can predict the feeding preferences of the target pollinator(s) of each species, and the nectar sugars (i.e., glucose, fructose, and sucrose) are a distinguishing feature of *Nicotiana* species phylogeny. Moreover, comparative statistical analysis indicate that pollinators are a stronger determinant of nectar composition than plant phylogeny.

## 1. Introduction

Nectars are metabolite-rich biological fluids that function as the primary floral reward offered to animal mutualists to sustain plant–pollinator relationships [1]. These fluids are produced and secreted from nectary glands. The nectar composition, in addition to other floral traits, such as morphology, color, and scent, recruits pollinators to promote plant reproductive success through improved pollination and seed set [2,3,4,5,6]. Collectively, these floral traits and nectar composition define the plant’s pollination syndrome, a complex trait that is thought to be evolutionarily optimized to accommodate the feeding preferences of the target pollinator [7,8,9]. 

Depending on the plant species, in addition to the major sugar components, which are primarily sucrose, glucose, and fructose nectar may contain amino acids, vitamins, alkaloids, phenolics, terpenoids, lipids, metal ions, hormones, and proteins [7,10,11,12]. The sugars and amino acids, which are the major classes of nectar metabolites, are thought to be especially influential in pollinator attraction [3,13,14]. The ratio of nectar sugars are a means of classifying nectars, into four categories: hexose-dominant, hexose-rich, sucrose-rich, and sucrose-dominant [7]. Sugar concentrations and compositional ratios are major determinants of nectar viscosity [15], which facilitates efficient feeding by pollinators such as honeybees, short tongue pollinators or hummingbirds [7,16,17]. Thus, honey bees and short tongue pollinators prefer concentrated hexose-rich nectars, whereas hummingbirds prefer sucrose-rich nectars [7,16,17]. Nectar viscosity is also impacted by nonsugar components, such as mucopolysaccharide, and these components are particularly important in rodent-pollinated systems [18]. As the second most common class of metabolites present in nectar, amino acids are typically a thousand-fold less abundant than sugars [12]. Amino acid composition also affects pollinator behavior and health [17,19,20,21]. For example, amino acids modulate the neuronal response of the pollinators and act as phagostimulatory metabolites (i.e., γ-aminobutyric acid (GABA), proline, ornithine and β-alanine) [9,22,23]. 

In this study we explored the correlations between floral nectar composition and other pollinator syndrome traits within *Nicotiana,* a genus which serves an important ecological model for plant–environment interactions [24,25,26,27]. These objectives were addressed by metabolomics analysis of the nectar from a broad sampling of fifteen *Nicotiana* species (*N.* spp.). These species represent both day- and night-flowering plants, and they are visited by five different pollinators (bees, butterflies, hawkmoths, moths, and hummingbirds).

## 2. Results

### 2.1. Nicotiana Floral Morphology and Pollination Syndromes 

**Figure 1:**
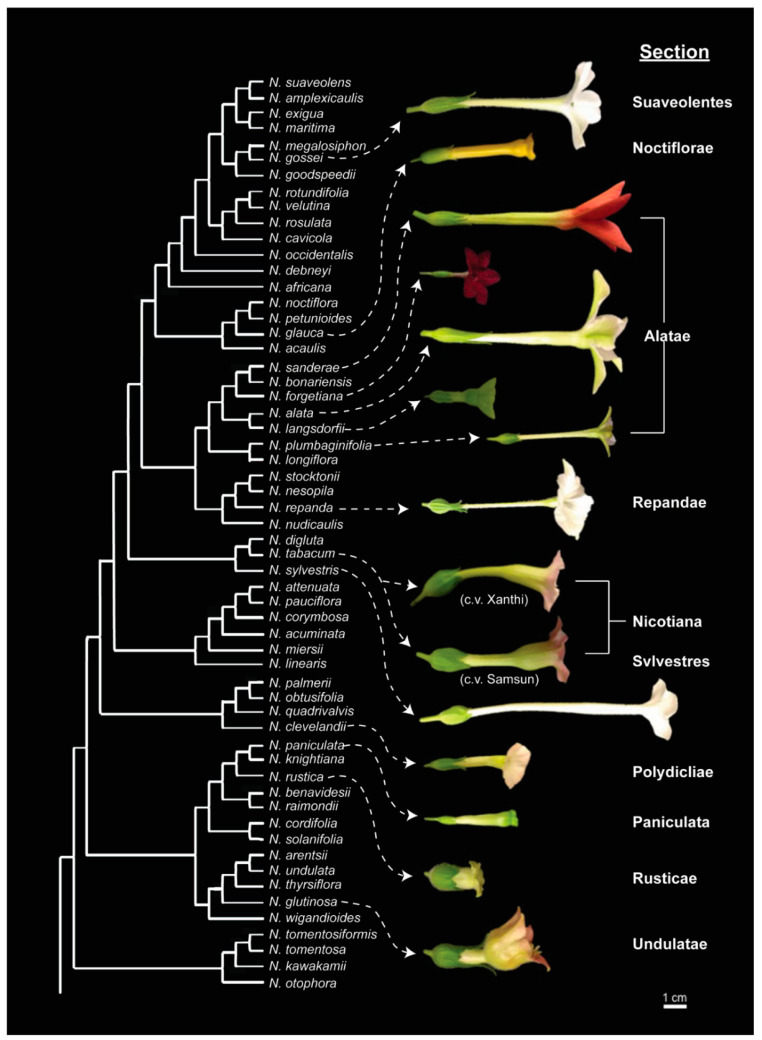
Flowers of the fifteen *Nicotiana* species studied herein, organized by phylogeny within the genus. Phylogenetic tree is based on the analysis of Clarkson et al. [27].

The pollination syndromes that attract the pollinators to *Nicotiana* are strongly influenced by floral morphology, flower timing, and nectar composition. The natural variation in these floral attributes was captured in this study by studying fifteen species representing ten sections within the genus. Morphological differences observed in *Nicotiana* flowers include petal color, floral opening width, and corolla length (Figure 1). The major pollinators that visit these species include bees, butterflies, hawkmoths, hummingbirds, and moths (Table 1).

### 2.2. Nectar Sugars and Amino Acids of Nicotiana Species

**Figure 2:**
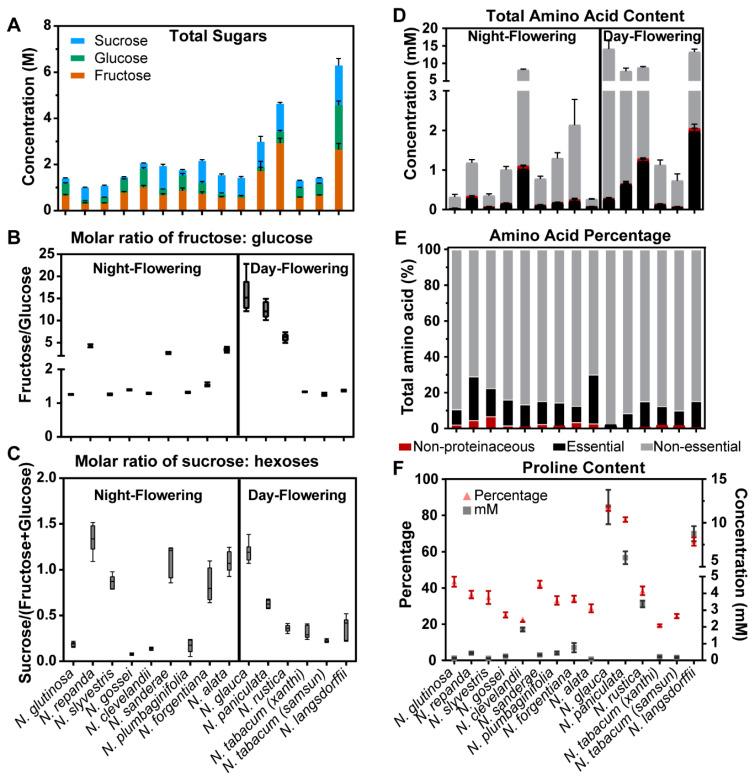
Sugar and amino acid profiles among the fifteen *Nicotiana* species. (**A**) Sum of total sugar content highlighting the contributions of each of the three main sugars, sucrose, glucose and fructose. (**B**) Molar ratio of fructose to glucose. (**C**) Molar ratio of sucrose to the total hexose content. (**D**) Sum of total amino acid content highlighting the contributions of nonproteinaceous, essential, and nonessential amino acid categories. (**E**) Proportion of nonproteinaceous, essential, and nonessential amino acid categories. (**F**) Percentage and concentration (mM) of proline in each nectar. Error bars represent the standard error, from 6 replicates.

The targeted gas chromatography–flame ionization detector (GC-FID) carbohydrate analysis of the predominant sugars in nectar (i.e., fructose, glucose, and sucrose) revealed a 6-fold range of total sugar concentrations, spanning from ~1 M for *Nicotiana repanda* to ~6 M for *Nicotiana langsdorffii* (Figure 2A; Table 1, Tables S1 and S2). Assessment of the sugar ratios reveals that the nectar of eight of the species are hexose-rich (sucrose:hexose ratio < 0.5), three are sucrose-rich (sucrose:hexose ratio 0.5 > 1), and the other four species produce nectars that are sucrose-dominant (sucrose:hexose ratio > 1) (Figure 2C). The sucrose: hexose ratios are highest in the night-flowering species, (*N. repanda*, *N. sanderae*, *N. forgentiana*, *N. alata* and *N. sylvestris*), with the one exception, the day-flowering species *N. glauca*. Species with the highest fructose-to-glucose ratios (ranging 4.5 to 16) (*N. glauca*, *N. paniculata*, and *N. rustica*) are all day-flowering species (Figure 2B; Table S2). 

The analysis of nectar amino acids by GC-MS identified 24 amino acids, nine of which are classified as essential for honeybees [14], six are nonproteinaceous, and the remaining are nonessential (Table S2). The total amino acid concentration in the nectar of the different species ranged from 0.25 mM to 14 mM (Figure 2D). The highest concentration of total amino acid content occurs in the nectar of four day-flowering species, *N. glauca* (14.1 mM), *N. langsdorffii* (13.2 mM), *N. rustica* (8.8 mM), *N. paniculata* (7.8 mM), and one night-flowering species *N. clevelandii* (8.2 mM) (Table S2). Among all fifteen species examined, the nonessential amino acids (alanine, glycine, serine, proline, asparagine, aspartic acid, glutamic acid, glutamine, and tyrosine) accounted for the largest proportion of the amino acids, ranging from ~70% to 98% (Figure 2E), and in all but two species, proline was the most abundant nonessential amino acid, ranging between 0.1 mM to 12 mM (accounting for ~20% to 84% of total amino acid content). The highest concentrations of amino acids occur in the nectar of the day-flowering species, *N. glauca* (11.8 mM, 84% total amino acid content), *N. langsdorffii* (8.7 mM) and *N. paniculata* (6.0 mM). The exception to this generalization is the nectar from *N. tabacum* and *N. clevelandii,* in which asparagine and glutamine dominate the respective amino acid pools (Figure 2F). The amino acid profiles of *N. langsdorffii* and *N. rustica* nectars are somewhat unusual, containing the highest concentration of phenylalanine (0.30 mM and 0.27 mM, respectively (Table S1); this nectar composition attribute is also associated with the very short flowers, which may also contribute to the pollination syndrome for these two species.

### 2.3. Nectar Composition of Nicotiana Sections 

**Figure 3:**
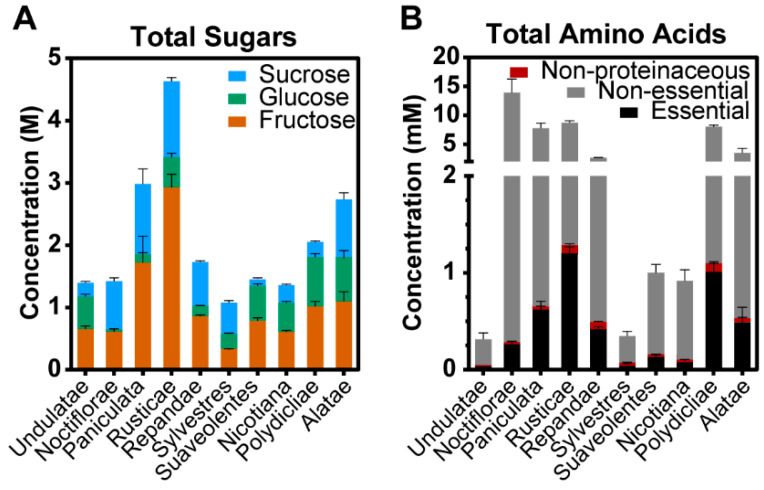
Sugar and amino acids profiles of ten *Nicotiana* Phylogenetic sections. (**A**) Sum of total sugar content highlighting the contributions of each of the three main sugars, sucrose, glucose and fructose. (**B**) Sum of total amino acid content categorized as nonproteinaceous, essential, and nonessential amino acids, Error bars represent the standard error, from 6 to 36 replicates depending on the section.

**Figure 4:**
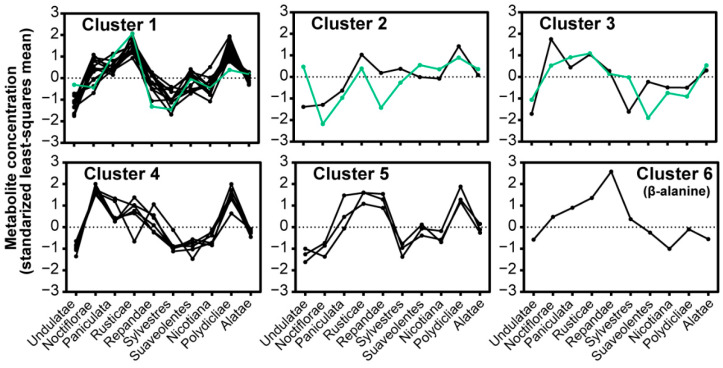
Hierarchical clustering analysis of the 27 quantified nectar metabolites grouped by *Nicotiana* Phylogenetic section. The three sugars are highlighted in green with fructose, glucose, and sucrose in Clusters 1, 2, and 3, respectively. Description of the amino acids within each cluster is provided in Table S3.

The relationship between nectar composition and the phylogenetics of the ten *Nicotiana* sections was evaluated by averaging the sum of the nectar sugar and amino acid profiles among the sections (Figure 3). Hierarchical clustering of these metabolomics data identified six distinguishable clusters (Figure 4; Table S3). These evaluations revealed unique nectar-defined features that are characteristic of the different *Nicotiana* section(s). A notable feature in this metabolite clustering analysis is the distribution of nectar sugars, which are statistically unique among eight of the ten *Nicotiana* sections; this conclusion is based on the *q*-values of pairwise comparisons between the sections (Figure 4; Table S3). *Nicotiana* sections with sugar profiles that are indistinguishable from each other are *Undulatae* and *Nicotiana* (Figure 3). 

The amino acid profiles of the *Nicotiana* sections provided further details to compare between the sections. The sections *Undulatae, Sylvestres, Suaveolentes*, and *Nicotiana* show equivalent amino acid profiles (Figure 3) with few significant differences in the abundances of the 27 quantified metabolites (Table S3). The similarity between these four sections is apparent in the patterns of metabolite abundances displayed in Clusters 1, 4, and 5 (Figure 4). The sections *Rusticae* and *Polydicliae* are similar as characterized by comparable proportions of amino acids when viewed by the functional category, i.e., nonproteinaceous, essential, and nonessential (Figure 3B), and parallel metabolite abundance patterns within Clusters 1 and 5 (Figure 4). The section *Noctiflorae* is distinguished from other sections by displaying significantly higher abundances of the six metabolites within Cluster 4, which includes proline and nonessential amino acids (Figure 4; Table S3). As illustrated by Cluster 6, the section *Repandae* is unique by the fact that the nectar contains high abundance of β-alanine (Figure 4). 

### 2.4. Relationship between Nectar Composition and Pollinators 

**Figure 5:**
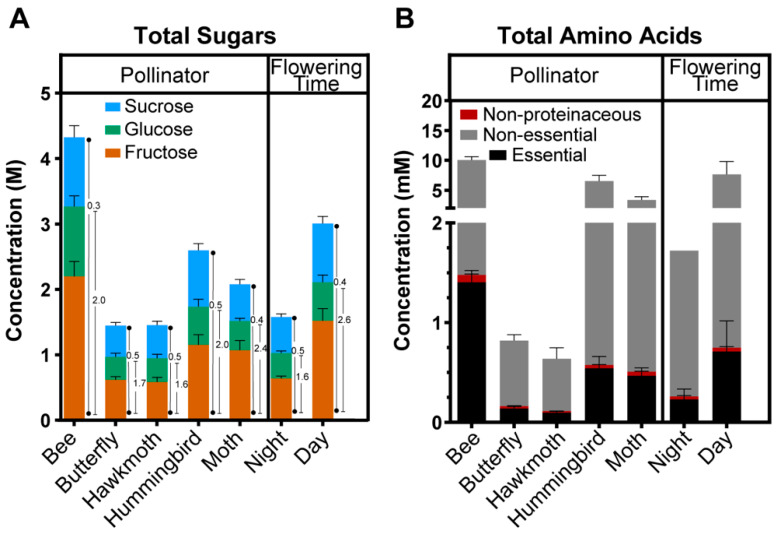
Sugar and amino acids profiles of *Nicotiana* nectars categorized by the preferred pollinator and time of flowering. (**A**) Sum of total sugar content highlighting the contributions of the three main sugars, sucrose, glucose and fructose. The numbers beside each data-bar is the sucrose: hexose molar ratio (closest to the data-bar) and the fructose: glucose ratio (furthest from data-bar). (**B**) Sum of total amino acid content in nectar categorized as nonproteinaceous, essential, and nonessential amino acids. Error bars represent the standard error, from 18 to 48 replicates depending on pollinator or flowering time.

**Figure 6:**
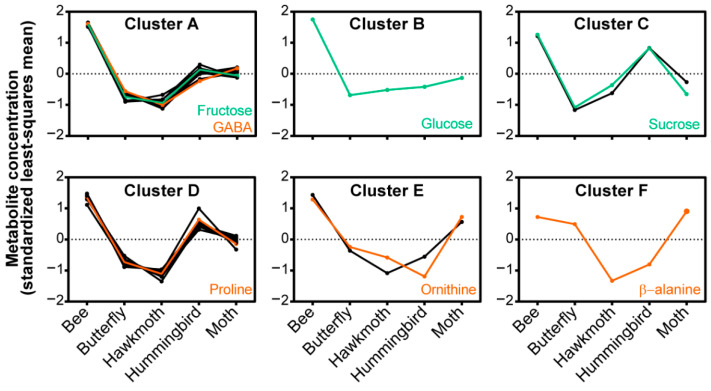
Nectar metabolites clustered by preferred pollinator. Hierarchical clustering analysis of the 27 quantified nectar metabolites grouped by preferred pollinator. Sugars are highlighted in green and phagostimulatory amino acids are highlighted in orange.

The relationship between nectar composition and the preferred pollinator that is attracted to the nectar of each *Nicotiana* species was also visualized by the average sum of the sugar and amino acid profiles (Figure 5) and by hierarchical clustering analysis. The latter analysis grouped the metabolite patterns into six distinct clusters (Clusters A–F) (Figure 6; Table S4). Nectars of species pollinated by bees are the richest in metabolite levels, with 4.3 M total sugars, 10 mM amino acids (Figure 5). The hierarchical clustering analysis also identifies that the bee-pollinated nectars show the highest abundance levels of metabolites in 5 of the 6 clusters, i.e., Clusters A–E (Figure 6). Moreover, 103 out of the 108 possible pairwise comparisons of metabolite abundances differed significantly between bees and all other pollinators (*q*-values < 0.05; Table S4). Individual analysis of amino acids that act as pollinator attractants [39,40] demonstrate that bee nectars have the highest levels of γ-aminobutyric acid (GABA), proline, and ornithine (orange highlights, Figure 6). 

Hummingbird-attracting nectars have the second highest total sugar and amino acid content, 2.6 M and 6.5 mM respectively (Figure 5). This trend is apparent in metabolite abundance patterns of Clusters A, C, and D (Figure 6). Nectars of moth-pollinated species appear to be intermediate in metabolite abundances, being less rich than the bee- and hummingbird-pollinated species, but richer than butterfly- and hawkmoth-pollinated species (Figure 5). As illustrated by the total amino acid profiles (Figure 3B) and patterns in Clusters A, B, and C (Figure 6), nectars of moth-pollinated species more closely resemble hummingbird-pollinated species (13 of 27 metabolite abundance patterns differ) than hawkmoth-pollinated species (24 of 27 metabolite abundance patterns differ) (Table S4). Distinguishing features of nectars of moth-pollinated nectars are the relatively higher abundances of lysine, ornithine, and β-alanine (Clusters E and F, Figure 6). 

The nectars that attract the remaining two pollinators, butterfly and hawkmoth, are characterized by feeding on nectars that are nearly identical in composition (Figure 5). These nectars generally contain the lowest sugar and amino acid metabolite abundances (Cluster A through Cluster D) (Figure 5). The abundances of only seven metabolites differed significantly between butterfly- and hawkmoth-feeding nectars (Table S4). 

**Figure 7:**
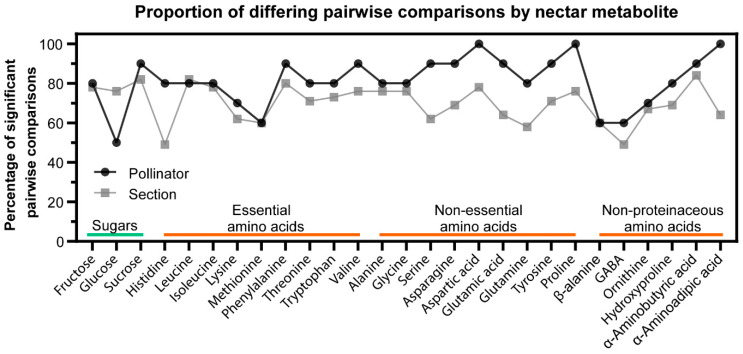
Proportion of significant pairwise comparisons (*q*-values < 0.05) for each *Nicotiana* nectar metabolite when summarized by phylogenetic section (grey, a total of 45 possible pairwise comparisons, Table S3) or preferred pollinator (black, a total of 10 possible pairwise comparisons, Table S4).

Figure 7 illustrates a statistical strategy to distinguish the role of plant phylogeny and the pollinator preference as determinants of nectar composition. Specifically, for each metabolite, we determined the proportion of significant concentration difference for each metabolite when summed by the preferred pollinator or the phylogenetic *Nicotiana* sections. For example, when evaluating proline, 76% (35 out of the 45) of possible pairwise comparisons among *Nicotiana* sections differed significantly, whereas 10 out of 10 (100%) of the possible pairwise comparisons differed among the pollinator preferences (Figure 7). Similar evaluations of all the metabolites demonstrate that there are more significant differences in nectar compositions among species that utilize separate pollinators when compared to the individual phylogenetic sections. Collectively therefore, these results suggest that pollinator preference is a stronger determinant of nectar composition (i.e., sugars and amino acids) than the phylogenic differences among the *Nicotiana* sections. 

## 3. Discussion

The primary objective of the current study was to define the influence of plant phylogeny and pollinator constraints on determining nectar composition. This was explored by leveraging the diversity in plant–animal interactions among *Nicotiana* species. The *Nicotiana* (Solanaceae) genus has adapted to wide ranging habitats, from deserts to subtropical regions distributed across South America, North America, Australia, the South Pacific, and Africa. Flowers in this genus are day-flowering or night-flowering [24,25] and are visited by several different pollinators [14,31]. Hence, these attributes make *Nicotiana* an ideal system for identification of ecological factors that may drive nectar composition and provide a basis to explore the variation in phylogenic relations, ecological conditions, flowering time, and pollination syndrome. 

Nectar is a complex secreted solution, which is predominately defined by the sugar and amino acid constituents. However, other minor constituents include vitamins, alkaloids, phenolics, terpenoids, lipids, metal ions, hormones, and proteins [7,11]. In the present study, we quantified nectar sugar and amino acid content from fifteen *Nicotiana* species, representing ten of the thirteen sections of the genus, and these species are pollinated by five different animals. This broad sampling provided a basis to assess the influence of multiple factors on nectar composition, specifically day/night flowering, phylogenetics of the plant, and the pollinators, and therefore explore how these traits interrelate to a particular pollination syndrome for *Nicotiana* [3,13,14].

Evaluation of the nectar composition at the species level confirmed that sugars are the predominant components, with total amino acid content accounting for about 0.1% molar mass of the nectar. Based on the current model of sugar nectar production, a one-to-one molar ratio of fructose-to-glucose is expected because these hexoses are thought to be generated by the hydrolysis of sucrose, catalyzed by an extracellular cell wall invertase [41,42]. In contrast to expectation, and consistent with prior findings [13,14], the fructose-to-glucose ratios display a nonstoichiometric hexose ratio skewed towards higher fructose levels. This skewed hexose ratio could be the result of postsecretion modification of the nectar caused by either in situ fermentation of the sugar by yeasts that are in the nectar [43], or the alteration of carbohydrate chemistries by enzymes secreted into the nectar [44,45]. As previously reported with closely related species [3,14], the most abundant amino acid is proline followed by glutamine, aspartic acid, and asparagine. 

### 3.1. Nicotiana Nectar Displays Compositional Differences Based on Flower Timing 

*Nicotiana* is comprised of both day-flowering and night-flowering species, and as previously indicated [14], the day-flowering species, *N. glauca*, *N. paniculata* and *N. rustica* produce nectars with a higher fructose-to-glucose ratio. This attribute may be associated with the unique metabolic capability of the predominant pollinator (i.e., hummingbirds) that visits these *Nicotiana* species, which can efficiently sustain flight with such hexose sugars. Namely, individually the sugars fructose and glucose respectively fuel 88% and 81% of the hummingbird’s metabolism during hovering flight [46]. Another nectar composition feature that is highly associated with the day-flowering species, is the high concentration of amino acids, particularly that of proline. 

The nectar composition of most night-flowering species (i.e., *N. repanda*, *N. sanderae*, *N. forgentiana*, *N. alata* and *N. sylvestris*; the exception being *N. glauca*), are dilute resulting in a less viscous nectar and have a higher sucrose: hexose ratio, which has the effect of decreasing osmolality. The lower viscosity of a dilute nectar aids the nocturnal pollinators (i.e., *Lepidoptera* moth), which feed by sucking nectar through a long proboscis [14,15]. Additionally, night-flowering plants can afford secreting a more dilute nectar solution, because the lower night-temperatures correlates with reduced rates of evaporation [47].

### 3.2. Plant Phylogeny and Pollinator Type Both Contribute to Nectar Composition 

Phylogenic variation within *Nicotiana* is reflected in the nectar compositions as illustrated in the hierarchical clustering of nectar sugar and amino acid profiles. Based on amino acid profiles, these analyses identified four groupings, one containing the sections *Undulataea, Sylvestres, Suaveolentes,* and *Nicotiana*, another containing *Rusticae* and *Polydicliae*, and two singular groups of *Noctiflorae* and *Repandae* sections. Thus, similar to the *Asteraceae* [2] and the *Labiatae* [48] families, nectar composition in *Nicotiana* has a strong phylogenetic determinant [3,14]. When only the nectar sugar profiles are considered however, a different phylogenetic grouping was obtained, with eight of the ten sections grouping separately (*Noctiflorae, Paniculatae, Rusticae, Repandae, Sylvestres, Suaveolentes, Polydicliae,* and *Alatae*) being distinguishable based on the significantly different combinations of fructose, glucose, and sucrose content. 

Kaczorowski et al. [3] suggested that floral nectar chemistry among *Alatae* species may be impacted by the pollinator’s feeding preference, a conclusion that was further supported by the study of Tiedge and Lohaus [14]. The nectar composition data presented herein appears to also support this conclusion, with distinct nectar profiles, which parallel the *Nicotiana* species pollinator feeding preferences (i.e., bee, butterfly, hawkmoth, hummingbird, or moth). It must be acknowledged that often a *Nicotiana* species is visited by multiple pollinators, and this complexity confounds the ability to correlate between pollinator preference and nectar composition. This potentially limits the accuracy of conclusions linking nectar composition to pollinator feeding preferences. For example, reflecting the known feeding preference of bees [7,22], bee-pollinated *Nicotiana* species (i.e., *N. rustica, N. clevelandii*, and *N. langsdorffii*) produce concentrated nectars that are hexose-rich, with a fructose-to-glucose ratio heavily skewed towards fructose. These bee-pollinated *Nicotiana* species also produce nectars that are rich in proline, phenylalanine, GABA, and ornithine. Generally, nectar amino acids are the major class of phagostimulatory metabolites and contribute to pollinator energy requirements [8,45,49]. These amino acids, particularly proline and phenylalanine, elicit a strong phagostimulatory response in bees [8,21,30,40,50,51]. Proline specifically provides rapid energy source for initial insect flight, which is particularly important for bees [39,52,53]. Artificial nectar enriched with GABA has been shown to increase the locomotion and survival of bees, particularly as compared to β-alanine enriched artificial nectar [49]. This maybe another explanation as to why nectar of bee-pollinated *Nicotiana* species are rich in GABA, but poor in β-alanine, the latter being rich in nectars of butterfly- and moth-pollinated species.

Hummingbird-pollinated *Nicotiana* species (i.e., *N. glauca, N. paniculata, N. tabacum, N. langsdorffii*, and *N. forgentiana*) produce sucrose-rich nectar, which aligns with the known hummingbird feeding preference [20]. Additionally, compared to nectars that attract bees, the sucrose concentrations are lower in these nectars, which aligns with the need for less viscous nectar to facilitate the feeding habits of hummingbirds, and deters the robbing of nectar by bees that require a higher viscosity nectar for “mopping tongue” feeding [14,15,54]. 

As nectarivores, moths are dependent on nectars for gaining the majority of their nutrient and energy needs. Similar to other moth-pollinated flowers [7], moth-pollinated *Nicotiana* species (i.e., *N. rustica, N. repanda, N. gossei, N. clevelandii*, and *N. sanderae*) produce a sucrose-rich nectar, which is also characterized by relatively high levels of the amino acids, lysine, ornithine, and β-alanine. 

The overall composition of the butterfly and hawkmoths nectars (i.e., *N. glutinosa, N. repanda, N. sylvestris, N. gossei, N. sanderae, N. plumbaginifolia,* and *N. alata*) were nearly identical and contain the lowest total sugar and amino acid abundances as compared to nectars preferred by moths, hummingbirds, and bees. One striking feature of these nectars is the high abundance of the nonproteinaceous amino acid, β-alanine. This nonproteinaceous amino acid is an insect neurotransmitter and may therefore enhance muscular endurance for prolonged flight [40]. Furthermore, this nectar-sourced β-alanine may provide the precursor that is required for melanin biosynthesis, specifically the biosynthesis of N-β-alanyl dopamine (NBAD) sclerostin, which is the biochemical basis for the yellowish-tan hues of butterfly wings [55]. 

Collectively, these data improve and expand upon prior studies of *Nicotiana* nectars ([3,13,14,31] and provide insights of the diversity in pollinator–nectar preferences and suggest that pollinator-mediated selection plays a critical role in the convergent evolution of different nectar types and floral diversification mechanisms. 

## 4. Materials and Methods 

### 4.1. Plant Materials and Growth Conditions

The seeds of fifteen *Nicotiana* species used in the study were obtained from the United States Department of Agriculture National Plant Germplasm System The seeds were sown in 4-inch peat pots in a greenhouse, cycling thru a 16 h illumination period, at a light level of 200 µmol·m^−2^·s^−1^, followed by an 8 h dark period, and temperature was maintained at 28 ± 5 °C. After 15 to 30 days, the seedlings were transplanted to individual 30 cm pots containing Sunshine® Mix #8 soil manufactured by Sun Gro Horticulture (Agawam, MA, USA). 

### 4.2. Nectar Sample Collection

Nectar was collected as previously described [56,57] between dawn and noon local time, from stage 12 flowers, the post maturation stage, when the flower is starting to open and the nectar starts to be secreted [58]. In brief, nectar was collected by separating the floral tube from the calyx of the flower and squeezing the base of the floral tube. Exuded nectar was collected using sterile micropipette tips (0.5–10 µL) and transferred to a 1.5 mL tube for long-term storage at −80 °C. During collection, nectar was kept on ice. A completely randomized experimental design was used during the nectar sample collection. The floral nectar, representative of each species, was sampled from three representative plants. Each nectar sampled was obtained by pooling nectar from three flowers of a plant, and two such samples were obtained per plant for a total of 90 samples (2 samples × 3 plants × 15 species). 

### 4.3. Amino Acid Analysis

Analysis of amino acids was performed using the EZ:Faast^TM^ kit for free amino acids (Phenomenex, Torrance, CA, USA). The nectar samples were subjected to solid phase extraction (20 µL nectar per extraction) and derivatization according to the manufacturer’s instructions, with one adjustment: after the addition of the norvaline internal standard (2 nmol), 170 µL of 10% propanol/20 mM HCl was added to acidify each sample. Following derivatization, samples were concentrated by evaporation under a stream of nitrogen gas, and amino acids were analyzed using an Agilent Technologies (Santa Clara, CA, USA) model 7890A gas chromatograph equipped with a ZB-AAA 10 m × 0.25 mm amino acid analysis column, coupled to a model 5975C mass selective detector capable of electric ionization. The gas chromatography-mass spectrometric (GC-MS) instrument settings followed the manufacturer’s recommendations. Analyte peaks were integrated using Automated Mass Spectral Deconvolution and Identification System (AMDIS) software package [59], and identified with authentic standards and the major ions listed by the manufacturer. Amino acids were quantified relative to the standard, norvaline, which was spiked into the isolated nectar at a concentration of 0.1 mM. 

### 4.4. Carbohydrate Analysis

Quantification of the predominant sugars (i.e., sucrose, glucose, and fructose) was completed by GC-flame ionization detector (FID) using an additional aliquot from the same nectar samples subjected to amino acid analysis. Specifically, 1 µL of nectar from the pooled sample was spiked with 10 µg ribitol as an internal standard, and the mixture was dried by lyophilization. The dried sample underwent methoximation while continuously shaking at 30 °C for 90 min, using 20 mg·mL^−1^ methoxyamine hydrochloride dissolved in pyridine. The methoximated sample was silylated for 30 min at 60 °C with BSTFA/ 1% TCMS. Following dilution with 1 mL pyridine, 1-µL aliquot was analyzed by GC-FID using an Agilent Technologies Model 7890A gas chromatograph system outfitted with an Agilent Technologies 7683B series injector and equipped with an DB-1ms (15 m × 250 µm × 0.25 µm) column coupled to an FID detector. 

Chromatography was conducted with a helium gas flow rate of 1.2 mL·min^−1^, and the injection was at 10:1 split-mode. The oven temperature gradient was in three steps, starting at 70 °C and increasing to 170 °C at a rate of 25 °C·min^−1^, then from 170 °C to 250 °C at a rate of 12.5 °C·min^−1^, and finally from 250 °C to 340 °C at a rate of 25 °C·min^−1^, followed by a 30 second hold at this temperature. Data integration of resulting spectra and analyte quantification were performed with the Agilent Technologies MSD ChemStation software Analyte peaks were identified by comparing retention times to authentic standards, and quantified using both the ribitol internal standard and calibration curves for each authentic sugar. 

### 4.5. Statistical Analysis

Statistical analysis of metabolite concentrations was conducted by taking the logarithm of the average values obtained from each plant, yielding 45 response values (one per plant and three per species). For each metabolite, a linear model with one mean per species and constant error variance was fitted to the 45 response values. As part of each linear model analysis, F-tests were conducted for each linear model comparing among the 15 *Nicotiana* species to identify differences in the average responses between each pair of *Nicotiana* sections and between each pair of pollinators. The 27 *p*-values for each comparison (one *p*-value per metabolite) were adjusted to obtain approximate control of the false discovery rate at the 0.05 level [60].

Similarities and differences among metabolite levels between *Nicotiana* sections were visualized by hierarchical agglomerative clustering with complete linkage. To perform clustering, the estimated section response means were first standardized within each metabolite to obtain a standardized response profile across sections for each metabolite. Dissimilarities between each pair of metabolites were computed as the Euclidean distance between the standardized response profiles. Clustering based on these pairwise dissimilarities places two metabolites in the same cluster if their estimated section response means are highly correlated among the *Nicotiana* sections. Although hierarchical clustering groups the metabolites into any number of clusters ranging from 1 to 27, a total of 6 clusters were selected for displaying and summarizing the results to strike a visual balance between “high within cluster consistency”, and “low between cluster similarity”. Using identical clustering methods, the metabolites were also clustered based on their estimated mean levels between different pollinators. 

## 5. Conclusions

Profiling the main classes of nectar metabolites (sugars and amino acids) among fifteen *Nicotiana* species identified that phylogeny within the genus and the pollinator syndrome traits such as the time of flowering and primary pollinators’ feeding preference are determinants of nectar composition. Collectively these data improve and expand upon prior studies of *Nicotiana* nectars [3,13,14,30] and provide insights on the diversity in pollinator-nectar preferences and suggest that pollinator-mediated selection plays a critical role in the convergent evolution of different nectar types and floral diversification mechanisms. 

## Figures and Tables

**Table 1:** Classification and characteristics of *Nicotiana* species (*N.* spp.) studied herein.

Species	Section	Preferred Pollinators	Flowering Time	Reference
*N. glutinosa*	*Undulataea*	Moth, butterflies	Night	[28]
*N. glauca*	*Noctiflorae*	Hummingbird, bird	Day	[14,29]
*N. paniculata*	*Paniculatae*	Hummingbird	Day	[14,30]
*N. rustica*	*Rusticae*	Moth, bee	Day	[31]
*N. repanda*	*Repandae*	Moth, butterflies	Night	[26,32]
*N. sylvestris*	*Sylvestres*	Hawkmoth	Night	[33]
*N. gossei*	*Suaveolentes*	Moth, butterflies	Night	[26,34]
*N. tabacum* (Xanthi)	*Nicotiana*	Hummingbird	Day	[14]
*N. tabacum* (Samsun)	*Nicotiana*	Hummingbird	Day	[14]
*N. clevelandii*	*Polydicliae*	Moth, bee, other	Night	[26,35]
*N. sanderae*	*Alatae*	Moths, butterflies	Night	[36,37]
*N. plumbaginifolia*	*Alatae*	Hawkmoth, autogamous	Night	[3]
*N. langsdorffii*	*Alatae*	Hummingbird, bee	Day	[3,38]
*N. forgentiana*	*Alatae*	Hummingbird	Night	[38]
*N. alata*	*Alatae*	Hawkmoth	Night	[3]

## Supplementary Materials

The following are available online at https://www.mdpi.com/2218-1989/10/5/214/s1, Table S1: Metabolite concentrations, Table S2: Summary *Nicotiana* floral nectar sugars and amino acids, Table S3: Hierarchical agglomerative clustering of Nicotiana nectar metabolomes by section, Table S4: Hierarchical agglomerative clustering of Nicotiana nectar metabolomes by pollinator. These supplemental files are held in the Iowa State public repository (https://iastate.figshare.com/), and this DOI will become available upon publication - 10.25380/iastate.7952615. Metabolomics data is publicly available in the PMR database (http://metnetweb.gdcb.iastate.edu/PMR/).
